# Society Meetings

**Published:** 1895-07

**Authors:** 


### Society Meetings.

The American Medical Publishers' Association held its second annual meeting at the Eutaw House, Baltimore, May 6 and 7, 1895, with the following in attendance: Dr. J. C. Culbertson, Cincinnati, Ohio; Miss Dora Jones, St. Louis, Mo.; Dr. John C. Le Grand, Anniston, Ala.; Dr. C. F. Taylor, William B. Saunders, Philadelphia, Pa.; Miss Hackedorn, Toledo, Ohio; Dr. F. E. Stewart, Detroit, Mich.; J. MacDonald, Jr., Irving J. Benjamin, Dr. Ferdinand King, Dr. H. P. Fairchild, New York City; Dr. R. W. Lowe, Bridgeport, Conn.; Dr. W. C. Wile, Danbury, Conn.; Dr. H. M. Simmons, Dr. Wm. B. Canfield, Baltimore, Md.; H. A. Mathie, Dr. A. H. Ohman-Dumesnil, Dr. I. N. Love, St. Louis, Mo.; Dr. Landon B. Edwards, Richmond, Va.; Dr. Hudson, Austin, Texas; Dr. William F. Bartlett, Philadelphia, Pa.; Dr. T. D. Crothers, Hartford, Conn.; Dr. Gilbert I. Cullen, Cincinnati, Ohio; Dr. Henry S. Upson, Cleveland, Ohio; Dr. E. E. Holt, Portland, Me.; J. M. Grosvenor, Jr., Boston; Charles Wood Fassett, St. Joseph, Mo.

Nineteen new members were admitted, and questions of the day affecting medical publishers were profitably discussed.

Beginning with July 1st, a monthly bulletin will be issued for the benefit of members of the association. It is to be edited by Drs. H. P. Fairchild, J. MacDonald, Jr., and Ferdinand King, New York City; Dr. J. C. LeGrand, of Anniston, Ala., and Charles Wood Fassett, of St. Joseph, Mo.

The secretary was authorized to issue, in pocket form, a revised list of medical advertisers.

The officers reëlected were as follows: President, Dr. Landon B. Edwards, of Richmond, Va.; vice-president, Dr. J. C. Culbertson, Cincinnati, Ohio; treasurer, J. MacDonald, Jr., New York City; secretary, Charles Wood Fassett, St. Joseph, Mo. Dr. J. C. LeGrand and Irving J. Benjamin were elected on the executive board.

---

A PERMANENT national organization of the various state medical examinining and licensing boards was effected during the American Medical Association meeting, May 9, 1895, at Baltimore, Md. Officers were elected for the ensuing year as follows: Dr. William Warren Potter, president, Buffalo, N. Y.; Dr. J. M. Hays, vice-president, Greensboro, N. C.; Dr. B. M. Griffith, secretary, Springfield, Ill. Dr. Charles McIntire, of Easton, Pa., was appointed chairman of a committee to draft a constitution and by-laws.

The purposes of the association are to aid in establishing a uniform schedule of requirements for all medical colleges and examining boards, and to assist in perfecting methods for higher medical education.

---

THE Mississippi Valley Medical Association will hold its twenty-first annual meeting at Detroit, Tuesday, Wednesday, Thursday and Friday, September 3, 4, 5 and 6, 1895, under the presidency of Dr. William N. Wishard, of Indianapolis. A circular has been issued by the secretary, Dr. Frederick C. Woodburn, inviting the medical profession to attend the meeting, and stating that titles of papers should be furnished at an early date. A large and enthusiastic meeting is anticipated.

---

THE Mitchell District Medical Society will hold its forty-seventh semi-annual meeting at West Baden Mineral Springs Hotel, West Baden, Orange County, Ind., on Friday and Saturday, July 5 and

6, 1895, under the presidency of Dr. Joseph Eastman, of Indianapolis. An interesting and extensive preliminary program has been published and the usual enthusiasm of these famous meetings promises to prevail.

---

THE American Association of Obstetricians and Gynecologists will hold its eighth annual meeting at Chicago, Tuesday, Wednesday and Thursday, September 24, 25 and 26, 1895, under the presidency of Dr. J. Henry Carstens, of Detroit. An enthusiastic meeting is already assured. The medical profession is cordially invited to attend the sessions.

---

THE American Electro-Therapeutic Association will hold its next annual meeting at Toronto, beginning September 3, 1895.

